# Thickness Effects for Thermoelectric Property of Antimony Telluride Nanoplatelets via Solvothermal Method

**DOI:** 10.1038/srep37722

**Published:** 2016-11-29

**Authors:** Xinxin Yan, Wenwen Zheng, Fengming Liu, Shuhua Yang, Ziyu Wang

**Affiliations:** 1 Department of Orthopaedic Surgery, Union Hospital, Tongji Medical College, Huazhong University of Science and Technology, Wuhan 430022, China; 2 Department of Orthopaedic Surgery, Wuhan Third Hospital, Wuhan 430060, China; 3 School of Science, Wuhan Institute of Technology, Wuhan 430205, China; 4 School of Science, Hubei University of Technology, Wuhan, 430068, China; 5 College of Physics and Electronic Science, Hubei Normal University, Huangshi 435002, China

## Abstract

Nanostructures have the potential to exhibit good thermoelectric properties by tuning and controlling their size and thickness, and the competing electrical and thermal properties can be decoupled by engineering the interface and grain boundary. In the present study, Sb_2_Te_3_ nanoplatelets with different sizes were fabricated using a practical solvothermal method. The thickness of the platelets were regulated between sizes of 10 nm and 100 nm, and the opposite edge length was varied between 1 and 10 μm by altering chemical conditions. Consequently, manipulating the grain size made it suitable to benefit the carrier transport and also block phonons for the thin platelets, resulting in a significant decrease in thermal conductivity and simultaneous increase in electrical conductivity. The results showed that the optimized figure of merit *ZT*, increased from 0.2 to 1.0 for thin samples, providing a comprehensive understanding of size-dependent thermoelectric performance.

In the past decades, global warming and the energy crisis have become increasingly severe and urgent. To tackle the challenges and risks these world problems pose, primary approaches include reduction of energy consumption and increased research on renewable and environmental energy^1^,^2^. Recently, particular interest and attention have been focused on low cost thermoelectric (TE) materials, which can be utilized for refrigeration and generation. The efficiency of thermoelectric materials is defined by the dimensionless figure of merit (*ZT*) and expressed as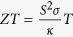, where *S*, *σ*, *κ* and *T* are the Seebeck coefficient, electrical conductivity, thermal conductivity, and absolute temperature, respectively. Electrical and thermal properties are two key factors that influence thermoelectric performance. Nevertheless, the three parameters (*S*, *σ*, *κ*) have strong trade-off between each other, where improvement in one parameter often leads to deterioration of the others and makes it more difficult to obtain high thermoelectric performance^3^. Nanostructuring has demonstrated to be an effective way to dramatically reduce thermal conductivity, which has minimal detrimental effects on electrical conductivity^4^,^5^,^6^.

The dominant effect of lowering thermal conductivity in nanostructuring materials is tuning the size and porosity of crystal grains^7^,^8^. The morphology and dimension of crystals play important roles in regulating transport of carriers and phonons^9^,^10^. When the grain size is comparable to the mean free paths of phonon, this part of phonon will be selectively scattered. According to the kinetic theory, thermal conductivity is calculated by the following expression: 
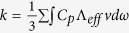
, where *C*_*p*_ is the specific heat capacity, 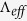 is the effective mean free path of phonon, *v* is the group velocity, and *ω* is the frequency of phonon. 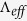 contains both the contribution from the bulk and the additional scattering part in grain boundaries, which obeys Matthiessen’ law, 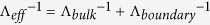. Grain boundary scattering is a critical scattering mechanism in blocking phonon transport of nanocrystalline TE materials^11^, except for umklapp scattering (phonon-phonon scattering) and impurity scattering. The effective mean free path for grain boundary scattering is closely related to the average grain size. Tailoring the grain size to optimize the transport of phonon is a crucial strategy for high thermoelectric materials^12^,^13^.

Sb_2_Te_3_ nanostructures are state-of-art p-type TE materials, which perform the maximum *ZT* value in the temperature range 300–500 K. The typical Sb_2_Te_3_ crystal consists of layered structures, where one layer is stacked by five atoms, Te^(1)^–Sb–Te^(2)^–Sb–Te^(1)^. Each layer is bonded through weak van der Waals interaction, so the cleavage plane is apt to occur perpendicular to the c-axis^14^. Owing to this unique rhombohedral crystal structure, it takes precedence to form two-dimensional nanostructures in Sb_2_Te_3_. Compared to various methods for fabricating TE materials with large grain size, such as hot pressing^15^, melt spinning^16^, and ball milling^17^, solvothermal technique is a practical and low cost bottom-up method used to synthesize nanostructured materials with controlled shape and size^18^. Solvothermal-synthesized Sb_2_Te_3_ is a hexagonal platelet-shaped nanostructures in which the size and thickness of the plate are controlled by an alkaline additive and surfactant agent. The nanoplatelets in diverse thickness and size behave different thermoelectric properties because the transport of electrons and phonons are strongly dependent on the grain thickness and size, which provides a facile and effective approach towards high thermoelectric performance via engineering the thickness of Sb_2_Te_3_ nanoplatelets.

Herein, Sb_2_Te_3_ nanoplatelets of different size and thickness were synthesized by modifying the solvothermal conditions. The thickness of hexagonal plates was tuned from 10 to 100 nm, and the edge length varied between 1 to 10 μm. The electrical conductivity and thermal conductivity showed strong dependence on the platelets thickness. Due to the size effect and boundary scattering, increase of electrical conductivity and reduction of thermal conductivity were simultaneously realized by controlling grain size, giving rise to enhancement of ZT value from 0.2 for the thick sample to 1.0 for the thin one.

Sb_2_Te_3_ nanostructures were synthesized via a reaction between antimony chloride and potassium tellurite in diethylene glycol solvent. The addition of the surfactant agent, polyvinyl pyrrolidone (PVP), results in varying the thickness of hexagonal nanoplatelets. The X-ray diffraction pattern (XRD) in Fig. 1 shows the phase structure of the Sb_2_Te_3_ nanostructures fabricated by this chemical route. The powders of nanoparticles with different thickness have similar diffraction peaks, which are indexed to be rhombohedral Sb_2_Te_3_ structures (JCPDS No. 15–0874). The well-structured crystalline correspond to the space group 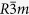.

In the process of redox reactions, PVP acts as a surfactant agent, which changes the grain size and thickness of products. PVP tends to preferentially attach onto the framework of growing Sb_2_Te_3_ particles, accelerating the speed that precursor precipitates from the solvent. As a result, the final products exhibit a large size and thick platelets in the presence of a certain amount of PVP. Owing to the inherently anisotropic crystalline structure of Sb_2_Te_3_, the cleavage plane slid perpendicular to the c-axis, resulting in the two-dimensional nanoplatelets. The size of the as-prepared Sb_2_Te_3_ nanoplatelets relies on the edge length and thickness of hexagonal plates. The scanning electron microscopy (SEM) image in Fig. 2 indicates that the Sb_2_Te_3_ nanoparticles are synthesized in different sizes. The thin hexagonal platelets are about 20 nm thick with a length of 1 μm between opposite edges, as shown in Fig. 2(a) and (b). Compared to thin grains, the average thick ones are observed to be about 8 μm in edge length and ~100 nm in thickness, as depicted in Fig. 2(c) and (d). The controllability of size provides the potential to optimize thermoelectric performance.

The obtained Sb_2_Te_3_ powders with different thickness are sintered into pellets by spark plasma sintering (SPS) to measure electrical and thermal property. The thermoelectric property of Sb_2_Te_3_ pellets with different thicknesses platelets are measured at a temperature range from 298 K to 523 K. The electrical conductivity of products with thin and thick particles as a dependent of temperature is shown in Fig. 3(a). All of the samples exhibit similar decrease tendency with the increase of temperature, revealing metal characteristics. The thin samples display higher electrical conductivity than the thick ones throughout the entire temperature range, which is contributed from the influence of size. The sintered samples with different thickness give rise to the change of carrier concentration and mobility. The thin sample perform optimized carrier transport, which is further studied through hall measurement and positron annihilation technique in the following text. The maximum electrical conductivity of thin platelets is 7.1 × 10^4^ S/m at room temperature, which outperforms thick platelets with 2.1 × 10^4^ S/m. The electrical conductivity of thin platelets is determined to be 3.8 × 10^4^ S/m at 523 K, which is higher than 1.29 × 10^4^ S/m of the thick ones.

The temperature dependence on Seebeck coefficient of the thick and thin platelets is shown in Fig. 3(b). The positive values of the Seebeck coefficient reveal that the fabricated Sb_2_Te_3_ semiconductor is p type, which is consistent with hall coefficient data in Table 1. All of the samples follow the same increasing trend with increase of temperature. There are not significant variations in Seebeck coefficients between the two specimens. The Seebeck coefficient is expressed as the following equation: 
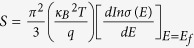
, where *κ*_*B*_ is Boltzmann constant, and *E*_*f*_ is the Fermi energy. The electronic structure and density of the state play important roles in determining Seebeck coefficient. The thickness of nanoplatelets make little contribution to changes in the band structure and Fermi energy, thus resulting in the value of Seebeck coefficient for the thin sample proximal to that of the thick one.

The power factor, calculated by S^2^σ, is shown in Fig. 3(c). Due to the increase in electrical conductivity with the rise of temperature offset by the decreasing trend in the Seebeck coefficient, the power factor of the two samples exhibit flat fluctuation over the entire temperature range. The power factor for the thin platelets is around 0.86~0.91 mW/(mK^2^), which is well above that of the thick platelets (0.27~0.3 mW/(mK^2^)), due to enhanced electrical conductivity.

The temperature dependence on thermal conductivity is shown in Fig. 3(d). When temperature is increased, the thermal conductivity of the two samples have a remarkable drop. Compared to the thick platelets, the thin ones display lower thermal conductivity (0.63–0.44 W/(mK)), which is also far below that of Sb_2_Te_3_-based bulk samples fabricated by melting^19^. The significantly reduced thermal conductivity of thin samples originates from the nanostructures. Spark plasma sintering (SPS) is a fast sintering and compaction process that is used to fabricate densified samples and prevent the growth of grains, therefore maintaining the nanostructured grains in Sb_2_Te_3_ pellets. The nanoparticles are arranged along the c-axis and stacked on top of others via pressure. As a consequence, the pellets stacked with thin platelets present more boundaries than those with thick platelets, eventually leading to more phonon scattering sites. It should be noted that boundary filtering plays an important role in forming barrier networks to block heat energy. Because of this, the thermal conductivity of the thin platelets is dramatically reduced. The *κ* value of thin platelets measured at 523 K is 0.44 W/(mK), which is reduced by 33% with respect to thick platelets.

To further explore the thermal conductivity of samples in different thickness, the experimental densities of the samples sintered by SPS and measured thermal diffusion coefficient are shown in Fig. 4. The density of Sb_2_Te_3_ samples with thick platelets is 4.7 g/cm^−3^, while that of the thin one is 5.2 g/cm^−3^. In the meantime, the obtained thermal diffusion coefficient is reduced from 0.523 (mm)^2^/s for the thick one to 0.372 (mm)^2^/s for the thin one. The different thickness of samples leads to distinct way of stacking crystals. In the process of spark plasma sintering (SPS), the powders with thick platelets tend to be sintered into a cylindrical pellet, preserving a lot of intervals between the large crystals, nevertheless, the thin platelets show less voids in the pellets. As a consequence, the density of Sb_2_Te_3_ pellets piled up by thin platelets is higher than that of the thick one, leading to a variation of thermal conductivity in different samples. Owing to the dense crystal arrangement in the samples with thin platelets, the boundaries are obviously increased, thus resulting in enhanced scattering phonons and reduced thermal conductivity.

Beyond boundary scattering, the contributing part of bipolar effect in thermal conductivity is also restrained in thin platelets, as compared to the thick one. The *κ*_*L*_ values of the samples are estimated using Wiedemann-Franz relationship *κ* = *κ*_*e*_ + *κ*_*L*_, where *κ*_*e*_ = *L*_0_*σT*, *L*_0_ is the Lorenz number, *σ* is electrical conductivity and *κ*_*e*_ is the electronic thermal conductivity, which is shown in Fig. 5. If *κ*_*L*_ is proportional to *T*^−1^ in the test temperature range, bipolar diffusion can be neglected. The difference existing in the fitted line and experimental *κ*_*L*_ value is the contribution from the bipolar diffusion effect^20^. As shown the significant “upturn” in lattice thermal conductivity of thick platelets indicate the bipolar thermal conductivity should not be ignored. In comparison, the linear change with *T*^−1^ for the lattice thermal conductivity of Sb_2_Te_3_ thin platelets reveals the little bipolar diffusion effect. This is another indicative for the decreased trend of thermal conductivity in the thin platelets sample.

These results indicate the thickness of the platelets influence the interface of SPS sintered pellets, determining the density of the samples. As a result, boundary scattering and bipolar diffusion effect varied in thin and thick platelets, making different contribution to the thermal conductivity. The sample of thin platelets perform lower thermal conductivity as compared to the thick one.

To further investigate the influence of carrier transport on electrical conductivity, hall measurements of thick and thin platelets are tested at room temperature to obtain the hall charge concentration and mobility, as shown in Table 1. The carrier concentration for the semiconductor is calculated by 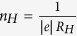, where *R*_*H*_ is the Hall coefficient. Subsequently, the carrier mobility is calculated from electrical conductivity and electron concentration measurements via 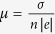. Both the carrier concentration and mobility simultaneously influence the electrical conductivity. The thick platelets with large grains, acting as phonon obstacles, led to a decrease in the carrier mobility from 113.583 cm^2^V^−1^s^−1^ for thin samples to 61.4198 cm^2^V^−1^s^−1^. The carrier concentration increase from 2.1529 × 10^19^ cm^−3^ for the thick platelets to 3.9429 × 10^19^ cm^−3^ for the thin ones. The vacancies and defects in samples vary with different thickness, contributing to the change in carrier concentration, which is further investigated by the technique of positron annihilation spectroscopy in the following text. Considering Hall measurement data, the simultaneous rise in carrier concentration and mobility result in the significant increase in electrical conductivity of Sb_2_Te_3_ thin platelets.

We further investigate the defects of the different samples via positron annihilation spectroscopy (PAS) to determine the influence of defects on thermal conductivity. Positrons carry a positive electronic charge, which can annihilate with an electronegative electron in the vicinity of vacancies and defects^21^. The variation of electron density influences the distribution of defects, and consequently, lifetime and intensity of positrons indicate corresponding defects and vacancies in a material^22^,^23^. Measurements of positron annihilation parameters offer a direct and effective tool to further analyze the effect of defects on electrical conductivity. By regulating the size of Sb_2_Te_3_ nanoparticles, the distribution of defects in pellets that are composed of thick and thin platelets shows large differences, resulting in size-dependent electrical conductivity. Figure 6(a) and (b) present the lifetime and intensity of Sb_2_Te_3_ thin platelets and thick platelets. The spectrum of thin samples are decomposed into two lifetimes, *τ*_1_ and *τ*_2_, with relative intensities, I_1_ and I_2_. In the thin sample, the positron lifetimes, *τ*_1_ and *τ*_2_, are 208.3 ps and 364.8 ps, which take up 50.3% and 49.7%, respectively. In comparison, the positron lifetimes, *τ*_1_ (217.7 ps) and *τ*_2_ (399.4 ps), occupied 50.8% and 49.2%, respectively, in the thick sample. The average lifetime, *τ*_*av*_, is calculated to comprehensively characterize the change in defects of different samples^24^. Considering the equation based on the positron parameters (lifetime and intensity), the average lifetime is calculated using the below expression: *τ*_*av*_ = *τ*_1_*I*_1_ + *τ*_2_*I*_2_. The acquired lifetime *τ*_*av*_ for the thin and thick platelets are determined to be 286.189 ps and 307.1.27 ps, respectively. The vacancies and defects in samples vary with different thickness, contributing to the change in carrier concentration. The vacancies in Sb_2_Te_3_ can easily trap positrons, resulting in electrons annihilating with positrons. The decrease of positron lifetime in thin platelets samples indicates more positrons annihilation, which are originated from vacancy-type defects located at the boundaries of grains. As the grain size of Sb_2_Te_3_ becoming smaller and thickness of platelets reducing, as-sintered Sb_2_Te_3_ pellets obtain more boundaries.

Rich vacancy defects exist in these boundary regions, which trap a lot of positrons to annihilate with electrons. As a consequence, the positron lifetime of thin platelets sample behave a decrease behavior as comparison with the thick one. These results demonstrate the higher vacancy defects in thin samples, which results in the increased concentration of carriers, as shown in Table 1. Associated with the increased carrier mobility, the optimized carrier concentration contributes to the increase of electrical conductivity. Additionally, it is noted that phonon scattering coming from the boundaries is reinforced in the thin platelets at the meantime. We contribute the significant boundary scattering to the decrease of thermal conductivity. This simultaneous increase of electrical conductivity and reduce of thermal conductivity in thin platelets samples indicate optimization of thermoelectric performance through tuning the thickness of samples.

The dimensionless figure of merit, *ZT*, for different thickness samples (Fig. 7) is increased as the temperature increase, which is contributed to the remarkable decrease in thermal conductivity with temperature. Considering decoupling electrical conductivity and thermal conductivity, the *ZT* value for thin platelets performs much higher than that for thick platelets. The maximum of the thin platelets is 1.012 at 523 K, which outperforms 0.21 of thick ones. This further reveals that thermoelectric performance is strongly dependent on grain size, and materials with nanostructures have obvious advantages over bulk. Precise control of thickness and size of chemically fabricated nanoparticles provide an important way to optimize TE performance.

In summary, we chemically synthesized Sb_2_Te_3_ nanoplatelets with different thickness in a practicable route. Subsequently, densified pellets were sintered using a fast SPS process and to investigate TE performance. The grain size and thickness had significant effects on electrical and thermal properties. Due to the simultaneous increase in carrier concentration and mobility of the thin sample, the electrical conductivity exhibited superior properties compared to the thick sample. Significantly, the decoupling of electrical conductivity and thermal conductivity in thin platelets was realized, which promoted *σ* to increase from 1.29 × 10^4^ to 3.8 × 10^4^ S/m at 523 K, while *κ* was reduced by 33% with respect to the thick platelets. We proposed that boundary scattering and inhibited bipolar diffusion effect was responsible for the remarkable decrease of thermal conductivity in thin platelets samples. As a result, the *ZT* value was optimized to 1.012 from 0.21 at 523 K by tuning the thickness and size of nanoparticles. This systemic investigation on size-dependent properties via engineering grain boundaries and interfaces offers a feasible approach to develop outstanding TE materials.

## Methods

The nanostructured Sb_2_Te_3_ was synthesized following a chemical polyol method in a solution of diethylene glycol (DEG) with addition of a stoichiometric of raw materials, K_2_TeO_3_ and SbCl_3_. Adjusting the amount of NaOH and surfactant agent polyvinylphrrolidone (PVP) prompted the final products to exhibit different sizes. The reaction occurred at 240 °C for 4 h, which formed a dark suspension. To remove the residual organic waste to prevent the insulated surfactant from interfering with electrical property, the suspending solution was washed by acetone and isopropyl alcohol several times. Afterwards, the as-prepared powders with different thickness platelets were sintered into pellets by spark plasma sintering (SPS). During the SPS process, a sintering temperature of 250 °C and pressure of 7 KN were exerted on the sealed products. Due to the fast heating and cooling rates, the samples were quickly compressed into cylindrical pellets without remarkable grain growth. Subsequently, the sintered specimens were separated into a rectangular bar and cubic chunk in order to obtain electrical and thermal property measurements, respectively.

## Measurements

The as-grown Sb_2_Te_3_ powder was placed on the sample table for characterization of phase structure via X-ray powder diffraction (XRD) using Cu Ka radiation. The diffraction peak was scanned from 20° to 80° at a speed of 4°/min. To investigate the microstructure and size of Sb_2_Te_3_ products synthesized in different conditions, field emission scanning electron microscopy (FESEM) was conducted on the sample, which was dispersed on silicon substrate by isopropyl alcohol. Furthermore, the curve of electrical conductivity and Seebeck coefficient were tested on a ULVAC ZEM-3 instrument in the temperature range, 298–523 K. In the meantime, the thermal diffusivity *D* for different samples was measured on a Netzsch LFA 457 system using a laser flash principle. After measurement of heat capacity *C*_*P*_ and density *ρ*, the thermal conductivity was calculated using the expression, *κ* = *ρDC*_*P*_. To analyze carrier concentration *n* and mobility *μ* of the samples, the Hall coefficient *R*_*H*_ was obtained on a Quantum Design PPMS apparatus. Positron annihilation spectroscopy is an efficient way to detect the defects via probing positron lifetime and intensity on Doppler broadening of annihilation radiation (DBAR) system.

## Additional Information

**How to cite this article**: Yan, X. *et al.* Thickness Effects for Thermoelectric Property of Antimony Telluride Nanoplatelets via Solvothermal Method. *Sci. Rep.* **6**, 37722; doi: 10.1038/srep37722 (2016).

**Publisher's note:** Springer Nature remains neutral with regard to jurisdictional claims in published maps and institutional affiliations.

## Figures and Tables

**Figure 1:**
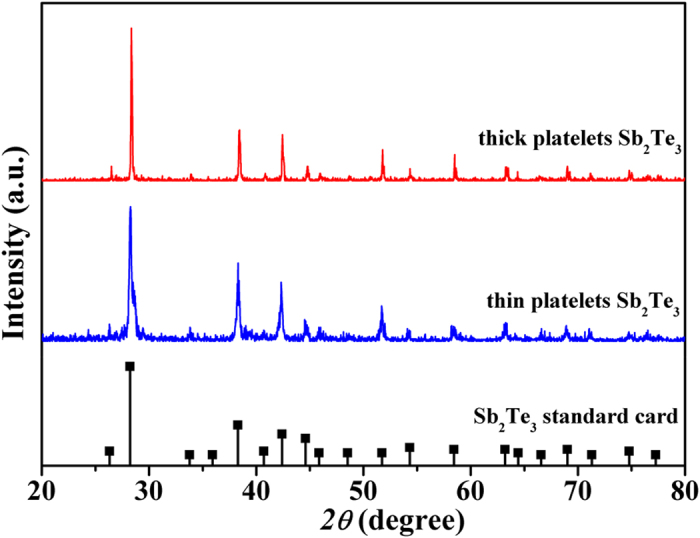
XRD patterns of Sb_2_Te_3_ nanostructured powder with different thickness.

**Figure 2:**
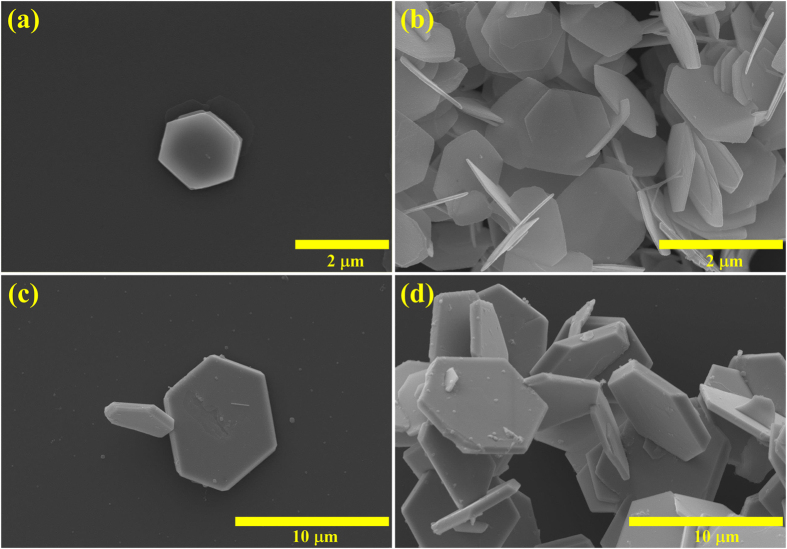
SEM image of the as-prepared Sb_2_Te_3_ nanoplatelets in thin platelets (**a**) and (**b**), and thick platelets (**c**) and (**d**).

**Figure 3:**
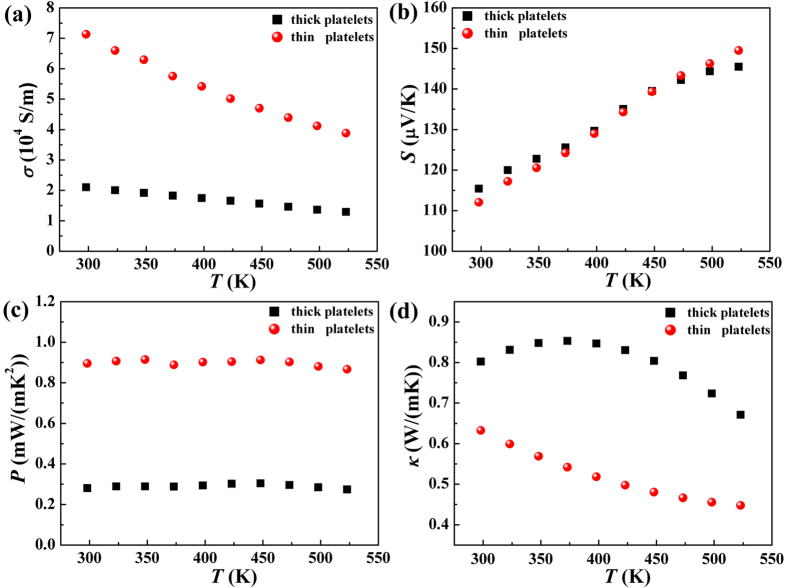
Temperature dependence of (**a**) electrical conductivity, (**b**) Seebeck coefficient, (**c**) power factor, and (**d**) thermal conductivity for Sb_2_Te_3_ samples with thick platelets and thin platelets.

**Figure 4:**
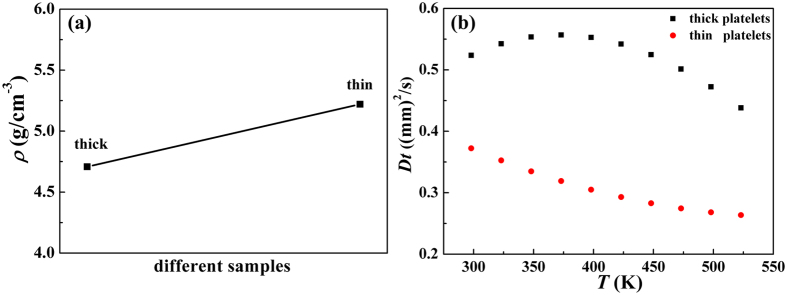
(**a**) The densities of the as-prepared different samples with thick and thin platelet. (**b**) Thermal diffusion coefficient of Sb_2_Te_3_ samples in thick and thin platelets.

**Figure 5:**
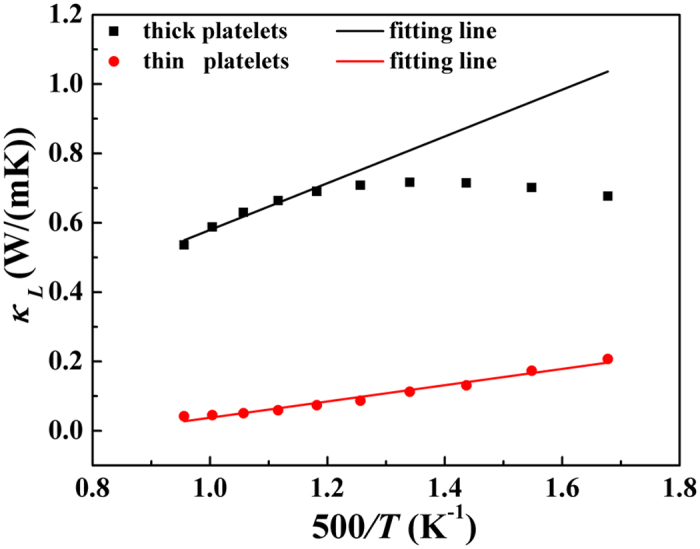
*T*^*−1*^ temperature dependence of lattice thermal conductivity *κ*_*L*_ for Sb_2_Te_3_ with thick and thin platelets. The scattered dots are the tested lattice thermal conductivity data and the solid line is the fitting line with the experimental data.

**Figure 6:**
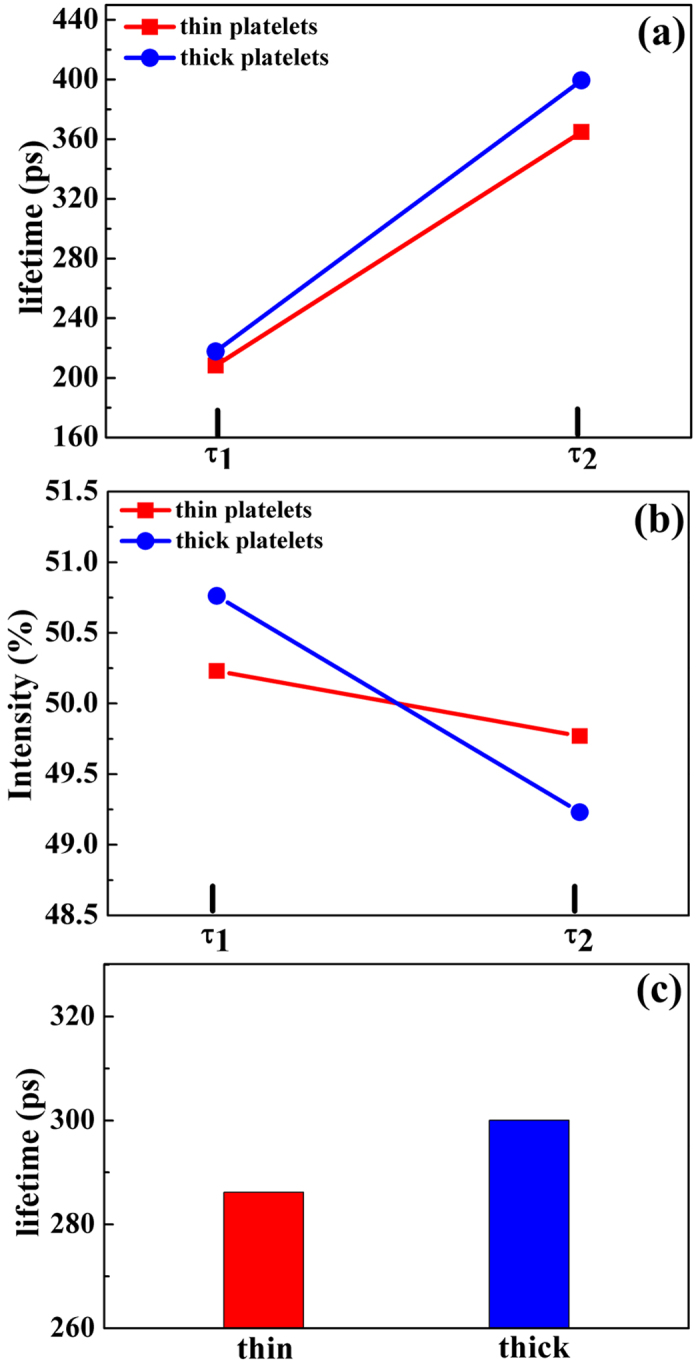
(**a**) The positron lifetime *τ*_1_, *τ*_2_ and (**b**) intensity *I*_1_, *I*_2_ for the fabricated thin and thick sample, and (**c**) the calculated average lifetime *τ*_*av*_ for different samples.

**Figure 7:**
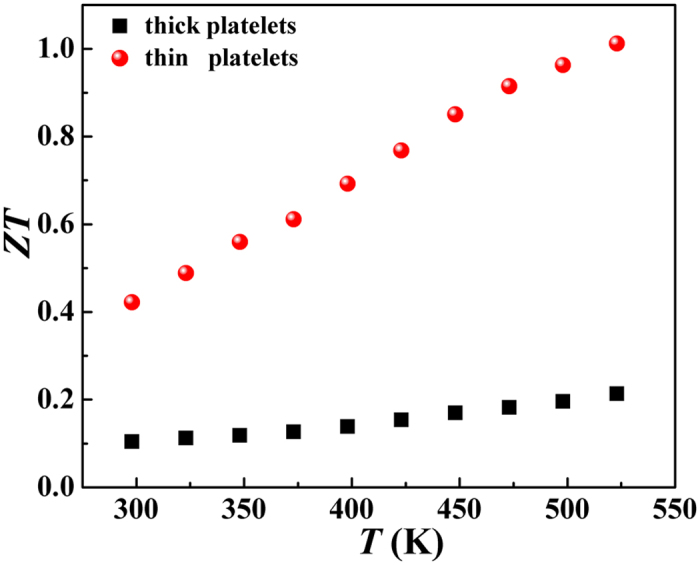
Temperature dependence of dimensionless figure of merit for Sb_2_Te_3_ thin and thick platelets.

**Table 1:** Hall measurement of Sb_2_Te_3_ samples with different thickness for carrier concentration and mobility.

	Hall coefficient (cm^3^/C)	Carrier Concentration (cm^−3^)	Carrier Mobility (cm^2^V^−1^s^−1^)
Thin platelets	0.1583	3.9429 × 10^19^	113.583
Thick platelets	0.28994	2.1529 × 10^19^	61.4198
